# Anesthesiology and cognitive impairment: a narrative review of current clinical literature

**DOI:** 10.1186/s12871-019-0903-7

**Published:** 2019-12-27

**Authors:** Jillian C. Belrose, Ruediger R. Noppens

**Affiliations:** Department of Anesthesia & Perioperative Medicine, Western University, London Health Sciences Center, 339 Windermere Rd, London, ON N6A 5A5 Canada

**Keywords:** Alzheimer’s disease, Dementia, Anesthesia, Postoperative cognitive dysfunction, Neurocognitive disorder, Elderly, Sevoflurane, Desflurane, Isoflurane, Propofol

## Abstract

**Background:**

The impact of general anesthesia on cognitive impairment is controversial and complex. A large body of evidence supports the association between exposure to surgery under general anesthesia and development of delayed neurocognitive recovery in a subset of patients. Existing literature continues to debate whether these short-term effects on cognition can be attributed to anesthetic agents themselves, or whether other variables are causative of the observed changes in cognition. Furthermore, there is conflicting data on the relationship between anesthesia exposure and the development of long-term neurocognitive disorders, or development of incident dementia in the patient population with normal preoperative cognitive function. Patients with pre-existing cognitive impairment present a unique set of anesthetic considerations, including potential medication interactions, challenges with cooperation during assessment and non-general anesthesia techniques, and the possibility that pre-existing cognitive impairment may impart a susceptibility to further cognitive dysfunction.

**Main body:**

This review highlights landmark and recent studies in the field, and explores potential mechanisms involved in perioperative cognitive disorders (also known as postoperative cognitive dysfunction, POCD). Specifically, we will review clinical and preclinical evidence which implicates alterations to tau protein, inflammation, calcium dysregulation, and mitochondrial dysfunction. As our population ages and the prevalence of Alzheimer’s disease and other forms of dementia continues to increase, we require a greater understanding of potential modifiable factors that impact perioperative cognitive impairment.

**Conclusions:**

Future research should aim to further characterize the associated risk factors and determine whether certain anesthetic approaches or other interventions may lower the potential risk which may be conferred by anesthesia and/or surgery in susceptible individuals.

## Background

A growing body of evidence has explored the whether exposure to anesthesia might cause temporary or long-term cognitive dysfunction. The specific impact of anesthesia on individuals with pre-existing cognitive impairment is also gaining attention. Dementia presents with impaired learning, memory, and reasoning. The worldwide prevalence of dementia in 2015 was 46.8 million, with a projected increase to 131.5 million by 2050 [1]. The estimated worldwide cost of dementia in 2015 was US$818 billion [2]. Dementia impacts length of hospital stay, morbidity, and mortality [3, 4]. Alzheimer’s disease (AD) accounts for 60–80% of dementia cases [3]. The projected increased prevalence suggests that anesthesiologists will be confronted with managing more patients diagnosed with AD and other forms of dementia. A greater understanding of the relationship between cognitive impairment with surgery and anesthesia is critical to guiding our clinical practice. This review will summarize key existing evidence in individuals with and without pre-existing cognitive impairment and will review potential pathways which may play a role in post-operative cognitive impairment.

## Main body

### Postoperative cognitive dysfunction

Postoperative cognitive impairment and the potential association with surgery under general anesthesia exposure was first described in 1955 [5]. Since this time, a substantial amount of research has been published which focuses on cognitive effects including delirium, postoperative cognitive dysfunction (POCD), development of dementia, and a decline in cognitive function in pre-existing dementia. POCD is often defined as a measurable impairment in cognition measured with neuropsychological testing in an individual over time, which may affect memory, attention, and psychomotor function [6]. In 2018, recommendations for nomenclature used to further define POCD were developed [7]. These definitions are summarized in Table 1. The implementation of more consistent terminology will allow for easier identification of trials assessing cognitive changes diagnosed up to 30 days after an operation (delayed neurocognitive recovery) vs. cognitive decline persisting beyond the 30-day recovery period (postoperative neurocognitive disorder). Due to variability in previous studies, the term POCD will be used interchangeably with the updated nomenclature throughout this review. In addition to updated nomenclature, the recent recommendations propose that screening cognitive tests are not sufficient for a diagnosis of a perioperative neurocognitive disorder, and instead diagnosis should involve assessment of performance on one or more cognitive domains. Prior literature has also exhibited wide variation in methodology employed to assess cognition, and differences in statistical analyses [6, 8–11]. The consequence of this is study heterogeneity which makes it challenging to draw definitive conclusions about various interventions or risk factors for perioperative neurocognitive disorders. Interestingly, individuals who self-report alterations in cognition postoperatively do not consistently demonstrate impairment on neuropsychological testing. Instead, some of these individuals may have higher levels of depression or anxiety [12]. This again highlights the need for the appropriate application of cognitive tests, and in the importance of considering a broad differential when patients report postoperative cognitive impairment.

**Table 1:** Nomenclature used for cognitive impairment at different perio-operative time periods

Time Period	Nomenclature	Definition
Preoperative	Mild Neurocognitive Disorder (NCD)	DSM-5 definition: (1) cognitive concern from the individual/informant/clinician + (2) objective evidence of decline of 1–2 SD compared to normative group + (3) maintained iADLs &/or ADLs
	Major NCD	DSM-5 definition: (1) cognitive concern from the individual/informant/clinician + (2) objective evidence of decline of ≥2 SD + (3) impaired iADLs &/or ADLs
Emergence	Emergence excitation or delirium	
After operation to postoperative day 30	Postoperative delirium	Fluctuating changes in attention, mental status, or level of consciousness which occur in hospital up to 1 week following surgery
	Delayed neurocognitive recovery	Cognitive decline meeting DSM-5 criteria for mild or major NCD, diagnosed within the 30 day recovery period
From expected recovery (30 days) to 12 months	Postoperative mild neurocognitive disorder (POCD) Postoperative major NCD (POCD)	Criteria as per DSM-5 for mild and major NCD Assumes decline cannot be accounted for by any other condition. Postoperative specifier implies temporal relationship. It does not imply causation. POCD is included as a specifier in parentheses while transitioning to the new nomenclature
Greater than 12 months postoperatively	Routine DSM-5 nomenclature	Postoperative specifier is NO LONGER attached if neurocognitive disorder is first diagnosed after this time.

The above nomenclature has been recently proposed to further define neurocognitive disorders associated with the perioperative period. Abbreviations: *DSM-5* diagnostic and statistical manual of mental disorders, *NCD* neurocognitive disorder, *SD* standard deviation, *iADL* instrumental activities of daily living, *ADL* activities of daily living; Objective evidence: tests of complex attention, executive function, learning and memory, language, perceptual-motor, or social cognition. Objective evidence cannot be limited to screening tools. This table is adapted from Evered et al. (2018) [7]

Initial research on POCD revealed that patients undergoing coronary artery bypass graft (CABG) procedures with cardiopulmonary bypass were more likely to develop intellectual dysfunction when compared with a similar subset of patients undergoing peripheral vascular surgery [13]. One week following cardiac surgery, cognitive decline is observed in 50–70% of patients [14]. Long lasting cognitive decline has also been observed, with 13–40% of individuals affected ≥1 year postoperatively [15–17]. One potential contributing factor to the high incidence of POCD in this population is the presence of microemboli from cardiopulmonary bypass [18]. In addition to microemboli, several other factors have been proposed, many of which are highlighted throughout this review. Interestingly, the progression of cerebrovascular disease itself in this patient population has also been proposed as a major contributor to the high incidence of neurocognitive disorder identified post-operatively. In support of this hypothesis, a prospective study comparing a group of patients undergoing CABG to patients undergoing medical management for CAD over a 6 year study demonstrated that patients in both groups showed a similar degree of cognitive decline over the study period [19].

The International Study of Post-Operative Cognitive Dysfunction (ISPOCD1) from 1998 increased interest in the association between surgery and perioperative neurocognitive disorders in non-cardiac surgery. The study enrolled 1218 patients aged 60 and older. Delayed neurocognitive recovery was present in 25% of patients 1 week after surgery, and postoperative neurocognitive disorder was present in 10% of patients 3 months after surgery [20]. Several risk factors for POCD were identified at the 1 week time point including age, level of education, duration of surgery/anesthesia exposure, a second operation, and postoperative infection or respiratory complications. At the 3 month time point, age was the only significant risk factor for POCD. Hypoxemia and hypotension were not identified as risk factors at either time point. Multiple trials have since been published to identify risk factors or interventions which can help to mitigate the potential impact on cognitive function.

### The effects of anesthetic agents on POCD

Several studies have aimed to determine whether general anesthesia itself is a risk factor for POCD. Many of these studies have chosen to compare general anesthesia (GA) to non-GA techniques such as neuraxial, regional, local anesthesia, and sedation. A meta-analysis published in 2010 looked at the existing literature on the topic. In their analysis, non-GA techniques included spinal, epidural, regional, and combination GA plus neuraxial or regional. POCD was defined by any objectively measured cognitive impairment. There was a non-significant trend toward increased POCD with GA vs. non-GA, with a 95% confidence interval (CI) of 0.93–1.95 [21]. Since the publication of this meta-analysis, a randomized controlled trial was published which compared GA to spinal without co-administration of confounding sedative medications. The trial population was patients ≥55 years of age undergoing extracorporeal shock wave lithotripsy. The authors concluded that the type of anesthesia did not impact rates of POCD at 7 days or 3 months postoperatively [22]. Of note, the trial did conduct an interim analysis and the trial was stopped early for futility at 50% of the a priori calculated required sample size. Furthermore, the mean age of the study population was 63.9 and 66.9 in the GA and spinal groups, respectively, and patients with pre-existing cognitive dysfunction were excluded from the study, which limits generalizability of the findings to individuals of advanced age and individuals with dementia. Indeed, the practice guidelines for perioperative brain health published in 2018 concluded that, based on current available data, there is insufficient evidence to recommend the use of regional anesthesia instead of general anesthesia [23].

Several studies have also been designed to assess whether the risk of POCD differs with general anesthesia using inhalational agents, vs. GA with TIVA (total intravenous anesthesia). A recent meta-analysis combined data from seven studies, and concluded that TIVA may reduce the risk of POCD, with an odds ratio (OR) of 0.52; however, this certainty of this conclusion is low due to heterogeneity of diagnostic tools utilized, variability in time of assessment, and inconsistent data reporting [24]. Furthermore, the authors reported that there were 11 ongoing studies on the topic at the time of the meta-analysis in 2018. At this time, it would be premature to conclude superiority of one mode of anesthetic.

The effect of depth of anesthesia on cognitive impairment has also been proposed as a potential risk factor for POCD. A meta-analysis recently compared cognitive outcomes in patients receiving low vs. high depth anesthesia as measured by bispectral index (BIS) monitoring. Included studies used either Propofol or Isoflurane. The authors concluded that depth of anesthesia did not significantly impact risk of POCD [25]. This conclusion was based on only 3 RCTs, and in one study the age of the participants was 45 ± 7.93 in the low BIS group, and 48.8 ± 10.2 in the high BIS group, which is unlikely to represent a patient population at risk for perioperative cognitive disorders. Furthermore, Hou and colleagues identified that there may have been variations in analgesic requirements between groups in the studies included in the meta-analysis which could confound results. Hou et al. (2018) proceeded to investigate patients aged 60 and older without pre-existing dementia scheduled to undergo elective total knee replacement. They randomized these patients to different depths of anesthesia as measured by BIS monitoring, with analgesic requirements controlled for using femoral and sciatic nerve blocks in both groups. Hemodynamic targets and induction doses were standardized. All patients received sevoflurane at 0.3MAC and a Propofol infusion titrated to target the appropriate BIS. On postoperative day 1, cognitive performance as measured by the Montreal cognitive assessment (MoCA) was significantly lower in the group with a target BIS of 40–50, when compared with the group with a target BIS of 55–65, indicating that a greater depth of anesthesia may increase POCD in the immediate postoperative period when other factors are carefully controlled [26]. There was no difference in cognition measured on postoperative days 3 or 7 which brings into questions whether a lower BIS has any meaningful clinical or economic significance. It should also be noted that the validity of BIS and other EEG-based depth of anesthesia monitoring does not correct for age or the presence of underlying cognitive dysfunction, and therefore may not serve as a reliable surrogate of depth of anesthesia in this patient population [27, 28]. Additional studies are required to validate depth of anesthesia monitoring in the elderly patient population, and also to investigate whether depth of anesthesia impacts the risk for cognitive impairment in the elderly population.

The effect of perioperative dexmedetomidine has been explored as a potential intervention to reduce the risk of POCD. The precise mechanism is still under investigation, but may relate to decreased requirements of other sedative and anesthetic medications, decreased opioids, modulation of the systemic stress response, promotion of natural sleep patterns, and potentially direct neuroprotective effects [29–31]. A landmark study published in 2016 demonstrated reduced delirium when low dose dexmedetomidine was administered postoperatively to elderly patients admitted to the intensive care unit following elective non-cardiac surgery [31]. The role of dexmedetomidine in prevention of POCD is less well studied, but has been the focus of a 2016 meta-analysis which concluded that dexmedetomidine may reduce the incidence of delayed neurocognitive recovery, and improve scores on the mini-mental state exam (MMSE) on postoperative day one [32]. This meta-analysis was limited by a small number of included studies with a small overall sample size, and study heterogeneity relating to patient inclusion and exclusion criteria and variations in dexmedetomidine administration. Since this time, many more studies have been designed to assess the impact of intraoperative and postoperative dexmedetomidine infusions in a variety of patient populations. Many of these studies are still underway. In the published literature, Deiner and colleagues (2017) randomized 404 elderly patients undergoing major elective non-cardiac surgery to dexmedetomidine infusion intraoperatively and 2 h postoperatively vs. placebo, with a primary outcome of delirium. A secondary outcome of cognitive impairment at 3 and 6 months postoperatively was also investigated. There were no differences in either delirium or cognitive performance; however, the study was designed and powered for the primary outcome of delirium, and the study was stopped for futility prior to reaching the calculated sample size of 706 [33]. Further high quality studies designed to assess cognitive function as the primary outcome are required to determine whether dexmedetomidine administration reduces the risk for perioperative neurocognitive disorders.

Cerebral perfusion and cerebral oxygen saturation have been implicated in the development of POCD. Chernov and colleagues (2006) utilized seven neuropsychological tests prior to and following CABG, and defined POCD as a 20% or greater reduction in score on 2 or more tests. Regional cerebral blood flow (rCBF) was measured with single photon emission computed tomography (SPECT) imaging. The study identified a relationship between reduced rCBF and cognitive performance [34]. Similarly, in patients who underwent CABG under hypothermic nonpulsatile cardiopulmonary bypass(CPB), a relationship was identified between reduced cognitive performance on the incidental memory assessment with cerebral blood flow measured by ^131^Xe clearance [35]. In another study, regional cerebral oxygen saturation was monitored with the INVOS cerebral oximeter in patients undergoing CABG. Patients were randomized to either a blinded control group or to an unblinded intervention group in which providers could intervene to improve cerebral oxygenation. Although there was no difference in cognitive decline between the groups, the study demonstrated that prolonged cerebral desaturation was associated with a higher risk of POCD [36]. Notably, there has also been research which has not replicated the relationship between cerebral blood flow and POCD. Specifically, Abildstrom and colleagues (2002) identified a global reduction in CBF following CABG using SPECT imaging, without any associated regional differences in CBF. In this study, there was no correlation between performance on neuropsychological testing and either global or regional CBF [37]. In the non-cardiac surgery population, a cohort study of elderly patients measured intraoperative cerebral autoregulation using bilateral transcranial doppler probes, and cerebral oxygenation using near-infrared spectroscopy (NIRS). Due to a large number of patients lost to follow-up, only the pre-operative and 1-week postoperative data was analyzed. The primary endpoint was assessed with a multivariable regression analysis, and did not identify an association between impaired intraoperative cerebral oxygenation or perfusion with POCD; however, a secondary analysis using a univariable logistic regression model did reveal a potential relationship between cerebral autoregulation and POCD [38]. Whether the potential association between cerebral perfusion or oxygenation with POCD is causative, or whether other confounds may explain the association observed is still unclear.

Limited available data also demonstrates mixed results regarding the impact of other modifiable factors on POCD risk including hypothermia [39, 40] and pre-operative and intraoperative glycemic control [41, 42]. Further prospective RCT evidence is required to evaluate the contribution of these factors to delayed neurocognitive recovery.

Although there is a need for further research and additional data to guide perioperative management of elderly individuals, recommendations have been developed based on current evidence [23]. The fifth international perioperative neurotoxicity working groups included over 30 experts who developed recommendations specific to postoperative brain health in individuals > 65 years of age. This article focused on pre-procedural consent, preoperative cognitive assessment, intraoperative management, and postoperative follow-up. We direct readers to the article by the working group for a more detailed description of their recommendations. Their recommendations are briefly summarized below:

(1) Consent: Individuals over the age of 65 should be informed of the risk of postoperative delirium and perioperative neurocognitive disorder prior to their procedure;

(2) Baseline cognitive assessment: Cognitive function should be assessed preoperatively using a brief screening tool. Examples of screening tools provided included the Minicog, MoCA, MMSE, clock-drawing test, verbal fluency test, or the cognitive disorder examination;

(3) Intraoperative management: Current literature is insufficient to define a specific anesthetic regimen to decrease risk of perioperative neurocognitive disorder; however, cautious use or avoidance of medications such as first-generation antihistamines, centrally acting anticholinergics, benzodiazepines, and meperidine was recommended. The authors also suggest avoiding relative hypotension, maintenance of normothermia, use age-adjusted minimal alveolar concentration (MAC) of volatile anesthetic agents, and use EEG-based depth of anesthesia monitoring to titrate anesthetic delivery.

(4) Postoperative follow-up: additional research is required to determine feasibility, efficacy, and cost-effectiveness of postoperative follow-up to assess cognitive outcomes [23].

In our opinion, the above recommendations reflect the evidence, as well as the continued uncertainty in the field. We do recognize that baseline cognitive assessment is often challenging to implement with limited time for preoperative assessment. Furthermore, without established postoperative follow-up, the utility of baseline cognitive assessment could be questioned. This highlights the need to establish assessments with strong reliability and validity which are relatively easy to administer, and which can be applied postoperatively to identify patients with impaired cognition in the perioperative period.

As summarized above, there is a large body of evidence which supports the existence of delayed neurocognitive recovery and postoperative neurocognitive disorder. Perioperative neurocognitive disorder is a distinct clinical presentation which is separate from Alzheimer’s disease and other forms of dementia. Based on our current knowledge, it is unclear whether the presence of delayed neurocognitive recovery or postoperative neurocognitive disorder confers an increased susceptibility to Alzheimer’s disease or other dementia. Indeed, the pathological mechanisms may be distinct from those involved in Alzheimer’s disease and other forms of dementia. Furthermore, it is currently unclear whether surgery and/or anesthesia increases the risk of subsequent diagnosis of dementia. A meta-analysis published in 2017 showed no significant association between GA and incident dementia (95% CI 0.90–1.19). Importantly, reporting bias may impact study results as patients may be unsure of the type of anesthesia they received. Indeed, a subgroup analysis did show a small but significant association between GA and risk of dementia when only studies using anesthesia records to collect exposure data were included (OR1.22; 95% CI 1.01–1.47) [43]. Since the publication of this metanalysis, a large prospective cohort study using data from the Korean National Health Insurance Service – National Sample Database was published which adds to the debate in the field [44]. The study followed patients 50 years of age and older without pre-existing dementia over a 12 year period and assessed for incident dementia as defined by an ICD-10 code of dementia and documented history of dementia medication. There were 44,956 individuals in the GA group, and 174,469 in the control group. The authors used a time-varying Cox hazard model to minimize time-dependent bias, and utilized propensity score matching to reduce potential confounding biases between groups. Exposure to GA was determined by a general anesthesia operation code in NHIS-NSC database. Individuals who did not have a general anesthesia operation code were assigned to the control group. Similar to prior studies, this study design would not be able to separate the effects of anesthesia exposure from the effects of surgical stress and other potential confounders (for example, perioperative hypothermia or administration of narcotics and other medications). After adjusting for important covariates, a multivariable survival analysis determined there was a 1.285-fold increased risk for developing dementia in the GA group when compared with controls [44]. The authors also conducted a multivariable analysis which demonstrated that the risk of incident dementia was increased with desflurane with a hazards ratio (HR) of 1.27, and isoflurane (HR 1.33), but was decreased with sevoflurane (HR 0.71) [44]. Although the difference hazards ratio of the inhalational agents is intriguing, the observational study design may include unaccounted for confounding variables which influenced choice of anesthetic agent, and independently increase the risk of dementia. A randomized controlled trial would be required to compare the risk profile of various anesthetic agents on cognition and risk for incident dementia. If a difference between volatile agents can be reproduced with a well designed RCT, further research would be required to explore the biological mechanisms differentially altered by various volatile agents.

In summary, it still unclear whether anesthesia itself causes POCD, or whether other surgical or patient factors might explain the observed cognitive impairment. Conflicting data also exists regarding the potential link between anesthesia exposure and risk of developing AD or other forms of dementia. Research assessing the risk profile of various anesthetic techniques is heterogeneous, and there is no strong evidence in favour of one technique over another. The debate in the field highlights the need for further high-quality research. Furthermore, whether there are additional considerations that should be applied to the individual with pre-existing cognitive impairment is unclear.

### Anesthesia in individuals with cognitive impairment

Alzheimer’s disease accounts for the majority of cases of dementia [3]. AD is a fatal progressive neurodegenerative disorder, characterized by neuronal degeneration in the basal forebrain, entorhinal cortex, hippocampus, and cortex [45–47]. Pathological hallmarks include the presence of senile plaques which contain Amyloid-β (Aβ), and neurofibrillary tangles which form in the presence of pathological modifications to the microtubule-associated protein, tau. The pathways underlying neurodegeneration are complex and involve many players including soluble and insoluble Aβ, hyperphosphorylated tau, neuroinflammation and microglia dysfunction, cholinergic deficits, and oxidative stress [48–53].

AD and other forms of dementia can impact the ability of anesthesiologists to collect a detailed history and elicit appropriate cooperation for physical examination. The potential for confusion and limited cooperation may make approaches such as neuraxial anesthesia, peripheral nerve blocks, or sedation more challenging. In terms of pharmacologic management, it is commonly accepted that short-acting medications should be used, and medications which may increase risk of postoperative confusion should be avoided [23, 54, 55]. Available evidence does not support the hypothesis that these patients are more sensitive to anesthetic agents; however, the sample size employed in these studies and use of the BIS monitor as a surrogate of anesthetic depth make it challenging to draw any definitive conclusion [27, 56].

Anesthesiologists must also be aware of drug-drug interactions, such as the interaction between neuromuscular blocking agents and acetylcholinesterase inhibitors [57, 58]. Donepezil (Aricept), a commonly prescribed medication in AD, is a reversible non-competitive cholinesterase inhibitor. It has a half-life of approximately 72 h, and requires 2–3 weeks for complete wash out [59]. Published case reports demonstrate that patients on donepezil may exhibit high intraoperative requirements for non-depolarizing neuromuscular blockers including atracurium, rocuronium, and vecuronium [57, 60]. In contrast, prolonged paralysis has been described with administration of succinylcholine to a patient on donepezil, potentially due to reduced pseudocholinesterase inhibitor activity [58]. In addition to these anesthetic considerations, it is also important to consider the potential long-term impact of anesthesia on cognition, morbidity, and mortality in patients with dementia.

The prevalence of preoperative dementia varies in different patient groups requiring surgery. For example, in the vascular surgery population, 3.8% of individuals have a pre-operative diagnosis of dementia [61]. In patients undergoing hip fracture surgery, an estimated 20% of individuals have a diagnosis of dementia [62]. In the vascular surgery population, dementia was identified as the strongest predictor for postoperative complications, increased hospital expenditures and was an independent predictor of increased mortality (HR 1.37) [61]. In a retrospective study, a multivariate analysis identified pre-existing dementia as an independent predictor of 30-day mortality following hip fracture surgery (*p* = 0.01) [63]. In a population-based retrospective cohort study, individuals with dementia who received GA were matched to similar patients receiving regional anesthesia. The type of anesthetic did not affect postoperative 30 day mortality, length of stay, or measured postoperative complications [64].

A meta-analysis published in 2014 concluded that there is an association between carrier status of the APOEε4 allele, a gene implicated in AD susceptibility, and risk of POCD one-week postoperatively [65]. This correlation was no longer present at 1–3 months or 1 year postoperatively [65]. A recent longitudinal cohort study assessed the interaction between gender and APOEε4 carrier status, and proposed that men carrying the APOEε4 allele have a more rapid decline in performance on tests of global cognitive function and memory when compared with women carrying the APOEε4 allele [66]. In individuals with pre-existing cognitive impairment, there is limited evidence to demonstrate whether exposure to anesthesia increases the risk of further cognitive decline. In one RCT, 180 patients with amnestic mild cognitive impairment (MCI) undergoing lumbar spinal surgery were randomized to sevoflurane, Propofol, or epidural analgesia. There was no difference in the risk of developing AD between groups after a 2 year follow-up; however, there was accelerated progression of amnestic MCI to progressive MCI in the group exposed to sevoflurane [67]. One study utilized data from 394 patients in the Oxford Project to Investigate Memory and Ageing (OPTIMA) cohort. Patients had at least two documented Cambridge Cognition Examination (CAMCOG) scores, had joined the study as controls or with MCI, and had been followed for at least 3 years. A total of 109 patients underwent surgery. Mixed-effects modeling was used to assess the association between CAMCOG scores and multiple variables, including exposure to surgery and anesthesia. The study identified a correlation between more rapid cognitive decline after surgery if cognitive impairment was diagnosed postoperatively (*p* = 0.0001) [68].

The preceding studies suggest that pre-existing cognitive impairment may impact postoperative morbidity, mortality, and/or health care costs. Furthermore, exposure to surgery and anesthesia may increase cognitive decline in these patients. Further research is required to better characterize this association, the resulting functional impact, and assess whether certain anesthetic approaches or other interventions can reduce the potential risk of morbidity in patients with pre-existing cognitive impairment.

### Potential mechanisms

In the clinical literature, it is challenging to isolate the contributions of surgical stress, various anesthetic options, and other confounding variables which may occur during the perioperative period. Therefore, elucidating mechanisms involved requires integration of knowledge gained from both the preclinical as well as the clinical literature. In this section, we will focus on key experiments which help us better understanding the multitude of factors which may be involved in development of POCD.

In animal studies, research has shown that surgery increases the risk of cognitive impairment when compared with exposure to anesthesia alone [69, 70]. One study established a model of abdominal surgery under local anesthesia alone to assess the effects of surgery without the confounding risk of sedative or anesthesia exposure. This group demonstrated that surgery alone, in the absence of a general anesthetic, produced cognitive impairment in 18 month old WT mice [71].

Exposure to anesthesia in the absence of a surgical stress does not reliably impair cognition when younger animals are utilized [72]. When aged animals are employed, there has been conflicting evidence assessing whether anesthesia exposure can lead to cognitive impairment [73–76]. The variability in results is likely impacted by heterogeneity in study design, including choice of animal models, age of animals, choice of anesthesia agent, dose, duration of exposure, and the choice of cognitive test and timing of assessment relative to anesthesia exposure. Despite the conflicting data on a single exposure, it does appear that repeat exposure to anesthesia may increase the risk for cognitive impairment in animal studies [77–79]. The use of transgenic animal models of Alzheimer’s disease also appears to increase the likelihood of cognitive impairment following surgery and anesthesia. This has been reviewed elsewhere [80]. Of interest, the timing of the exposure relative to the disease process may be important. For example, one study demonstrated that isoflurane exposure produced cognitive impairment in wild type (WT) mice at 12 months of age, but not in the Tg2576 AD mouse model at the same age [81]. It has been postulated that this may because the disease process was advanced in the AD animal model, and further impairment could not be induced with exposure to anesthesia. Furthermore, cognitive impairments were also not observed in 3xTgAD mice exposed to isoflurane at a 2, 4, or 6 months of age, prior to onset of pathology [82].

One hypothesis underlying the mechanism for post-operative cognitive impairment is that exposure to surgery and/or anesthesia will produce changes in Aβ and tau levels which mirror the changes observed in patients with MCI and AD. To examine this hypothesis, we will first review some evidence on antemortem biomarkers observed in AD and MCI. In patients with AD, several studies have demonstrated an increase in Total Tau (T-Tau), phosphorylated Tau (pTau), and neurofilament light levels in cerebral spinal fluid (CSF), and a decrease in CSF Aβ_1–42_ [83]. In plasma, concentrations of Aβ to not appear to be associated with the diagnosis of AD or the severity of cognitive impairment measured by MMSE [84, 85]. The ratio of Aβ_1–40_ / Aβ_1–42_ may be more reliable than the absolute concentration of these markers independently at predicting progression from MCI to AD, or differentiating early onset sporadic AD from age-matched controls [86, 87]. In a recent meta-analysis, only an increase in plasma T-tau could be used to differentiate between control and AD plasma samples (AD:control ratio of 1.95) [83].

There is a small amount of evidence exploring the effects of anesthesia on these biomarkers in individuals without pre-existing cognitive impairment. In one study, 11 patients without cognitive impairment were recruited who were scheduled to undergo endoscopic nasal surgery to correct idiopathic CSF leaks. The procedure requires placement of a lumbar drain which is left in situ for 24–48 h, or until accidental extradural placement. Over a 24-h period following surgery, there was no change in CSF Aβ_1–42_ concentration; however, there was an increase in the CSF concentration of total tau by more than 200%, and an increase in levels of pTau^181^ [88]. In a subsequent study, patients undergoing procedures that required the placement of a lumbar drain were randomized to either inhalational anesthesia with isoflurane or to TIVA using Propofol for maintenance [89]. The authors found no changes in CSF Aβ_1–42_ levels but did identify an increase in total tau levels 24 h post-operatively when compared with the time of drain insertion. In contrast to prior research, this study identified a decrease in pTau over time [89]. There was no interaction between the type of anesthesia employed and the biomarker levels in CSF; however, the authors appropriately point out that all patients underwent surgical manipulation of a dura and blood brain barrier, and this confound may outweigh any potential effects from the choice of anesthesia technique. Of note, this study did not examine the effects of isoflurane or propofol on cognition postoperatively Furthermore, neither this study nor the 2011 study mentioned whether a phosphatase inhibitor was included in the CSF at the time of collection, which may make any alterations in level of phosphorylated proteins less reliable, and/or more difficult to interpret. Pikwer and colleagues (2017) did not identify a changes in CSF Aβ or tau levels with a Propofol and remifentanil based TIVA; however, the mean age in this study was 50 years, and the follow-up time for CSF analysis was at 0, 2, and 5 h following spinal catheter placement, which raises the question of whether an older study population or later timepoint may have demonstrated changes in biomarker levels [90]. Separate studies have also examined whether preoperative biomarkers predict development of delirium or POCD. Indeed, plasma levels of Aβ_1–42_ were significantly lower in patients scheduled for CABG who developed POCD [15]. In another study, CSF was collected during spinal anesthesia for total hip or knee replacement. Lower CSF Aβ/Tau ratios were associated with a higher incidence of delirium as measured by the confusion assessment method and memorial delirium assessment scale [91].

These same pathways have also been explored in preclinical studies using animal models. In one landmark paper, Planel and colleagues (2007) demonstrated that tau hyperphosphorylation occurred when anesthesia was induced with either chloral hydrate or isoflurane. Interestingly, both of these agents also cause hypothermia, and maintenance of normothermic conditions was able to attenuate the hyperphosphorylation of tau [92]. In a separate study, maintenance of normothermia was shown to abolish tau hyperphosphorylation following exposure to anesthesia in aged mice, and also attenuated cognitive impairment in the animals [93]. Although these papers show that maintenance of normothermia is an important consideration, there are likely several other factors involved. Indeed, when animal models of AD are employed, exposure to anesthetic agents promotes tau hyperphosphorylation even when normothermia is maintained [82, 94–97]. This suggests that the susceptible brain may be more prone to pathological changes following exposure to anesthesia and/or surgery. In support of the link between pathologic modifications to tau and development of POCD, animal studies have shown that modulation of tau pathology using transgenic approaches or pharmacological approaches may decrease the risk of cognitive impairment following anesthesia and surgery [98, 99].

Inflammation has also been associated with POCD. An increase in various cytokines including IL-6, TNF-α, IL-8, and IL-10 have been correlated with postoperative cognitive impairment [88, 100, 101]. Recently, a meta-analysis was conducted which assessed for the association between various inflammatory biomarkers and POCD. The meta-analysis found that higher post-operative c-reactive protein (*n* = 11 studies) and interleukin-6 (IL-6) (*n* = 17 studies) are associated with POCD [102]. In the endoscopic nasal surgery study mentioned previously, the authors compared CSF from patients receiving anesthesia with sevoflurane to those who received TIVA and noted a significant increase in postoperative concentration of IL-6 in the patients receiving sevoflurane. Importantly, the study is limited by a small sample size and the fact that anesthetic approach was not randomized [88]. The increase in inflammatory markers has also been correlated with an increase in high rates of insulin resistance as calculated by the homeostasis model assessment 2, raising the possibility of a potential combined impact of inflammation and insulin resistance with development of POCD [100].

In animal studies, there is mixed evidence regarding whether volatile agents increase pro-inflammatory cytokines and/or microglial activation in the absence of surgery. Although an increase in inflammation has been observed by some groups [75, 103–105], other groups have reported no effect of isoflurane or sevoflurane anesthesia alone on microglial activation and/or proinflammatory cytokine expression [70, 82, 106, 107]. The mechanism for induction of neuroinflammation following exposure to anesthesia with or without surgery continues to be actively investigated. Potential factors involved include P2X7 receptors, NFκB, and increased blood brain barrier permeability [107–109].

Significant preclinical data has also explored the effects of anesthetic agents on synapse structure and function and calcium homeostasis. For example, volatile anesthetic agents, with or without surgery, have been shown to alter expression of post-synaptic density 95 (PSD-95) [73, 110–112]. Exposure to volatile agents has also been shown to impact expression of the extrasynaptic N-methyl-D-aspartate (NMDA) receptor GluN2B subunit, trafficking of the GluA1 subunit of the α-amino-3-hydroxy-5-methyl-4-isoxazoleproprionic acid (AMPA) receptor, and expression of the α5-γ-Aminobutyric acid A (α5GABA_A_) receptor [113–115]. Electrophysiological assessment of synaptic plasticity following exposure to volatile agents has also demonstrated impaired long-term potentiation and facilitated long-term depression [114, 116]. A summary of the potential role of calcium dysregulation downstream of anesthetic exposure has also previously been published [117]. In vivo preclinical evidence suggesting that calcium dysregulation participates in anesthesia-mediated neurotoxicity includes the finding that nimodipine, an L-type calcium channel antagonist, protects against sevoflurane or isoflurane-induced cognitive deficits, neuroinflammation, and apoptosis [103, 118, 119]. Inhibition of the NMDA receptor with memantine has also been shown to protect against the isoflurane-mediated increase in cytosolic calcium, caspase-3 activation and cytotoxicity in vitro [120].

Preclinical research has also focused on mitochondrial dysfunction and oxidative stress as a potential contributor to POCD. To examine this hypothesis, one study used animals with a knockout of Cyclophilin D (CypD), a protein which contributes to opening of the mitochondrial permeability transition pores. Knockout of CypD improves mitochondrial function and stability. These animals were crossed with an Alzheimer’s disease mouse model. Knockout of CypD resulted in improved cognitive performance following exposure to sevoflurane and laparotomy when compared with AD transgenic mice exposed to the same conditions [112]. Pharmacological targeting of the pathway with SS-31 pre-treatment, a mitochondrial targeted peptide anti-oxidant, similarly attenuates cognitive deficits and biochemical changes induced by a 2 hour exposure to isoflurane in aged mice [73].

In summary, several molecular pathways likely participate in the development of POCD. Clinical and preclinical evidence points toward a possible role for pathological modifications to tau, as well as neuroinflammation. Preclinical data also suggests that altered synapse structure or function, calcium dysregulation, and mitochondrial dysfunction may participate in the development of cognitive impairment following exposure to anesthesia and/or surgery. Elucidation of pathways upstream and downstream of these observed changes are under active investigation.

## Conclusions

This review summarizes previous findings relevant to the potential relationship between surgery, anesthesia exposure and POCD. AD or similar underlying pathology may make individuals more susceptible to the potential neurotoxic effects of the surgical stress and/or anesthesia exposure and increase the risk of progression of cognitive impairment. Several mechanisms have been implicated including altered Aβ processing or accumulation, pathological modifications to tau, neuroinflammation, calcium dysregulation, and mitochondrial dysfunction.

Review of the literature identifies several areas which require further investigation. First, additional high-quality clinical studies are required which focus on individuals with a pre-operative diagnosis of MCI, AD or other forms of dementia. Consistency in the evaluation of anesthesia exposure and measurement of cognitive function will be an asset to determine the true effect of anesthesia exposure. Second, well controlled RCTs are required to determine whether particular agents or anesthetic approaches have a more favorable risk profile. Third, additional clinical work and basic science research investigating molecular mechanisms at play will help to identify susceptible individuals, and possible interventions which could eventually be incorporated into clinical practice to minimize the risk of POCD. With the expected increase in AD prevalence and associated cost, and in the absence of effective disease modifying therapeutic agents to treat AD, clinicians and scientists must aim to identify and modify factors which may contribute to cognitive impairment in this subset of patients.

## Data Availability

Narrative review: not applicable.

## Abbreviations

AD: Alzheimer’s disease
AMPA: α-amino-3-hydroxy-5-methyl-4-isoxazoleproprionic acid
Aβ: Amyloid-β
BIS: Bispectral index
CABG: Coronary artery bypass graft
CAMCOG: Cambridge Cognition Examination
CI: Confidence interval
CPB: Cardiopulmonary bypass
CSF: Cerebral spinal fluid
CypD: Cyclophilin D
GA: General anesthesia
HR: Hazards ratio
IL: Interleukin
MAC: Minimal alveolar concentration
MCI: Mild cognitive impairment
MMSE: Mini-mental state exam
MoCA: Montreal cognitive assessment
NIRS: Near-infrared spectroscopy
NMDA: N-methyl-D-aspartate
OR: Odds ratio
POCD: Postoperative cognitive dysfunction
PSD-95: Post-synaptic density 95
pTau: Phosphorylated tau
rCBF: Regional cerebral blood flow
SPECT: Single photon emission computed tomography
TIVA: Total intravenous anesthesia
T-Tau: Total tau
WT: Wild type
α5GABA_A_: α5-γ-Aminobutyric acid A

## References

[1] Prince M, Wimo A, Guerchet M, Ali GC, Wu Y-T, Prina M. World Alzheimer Report 2015, The Global Impact of Dementia: An analysis of prevalence, incidence, cost and trends. Alzheimer’s Dis Int ADI Lond. 2015;87. https://www.alz.co.uk/research/world-report-2015. (last accessed 10/2018).

[2] Wimo A, Guerchet M, Ali G-C, Wu Y-T, Prina AM, Winblad B (2017). The worldwide costs of dementia 2015 and comparisons with 2010. Alzheimers Dement J Alzheimers Assoc.

[3] Alzheimer’s Association (2018). 2018 Alzheimer’s disease facts and figures. Alzheimers Dement J Alzheimers Assoc.

[4] Möllers Tobias, Stocker Hannah, Wei Wenjia, Perna Laura, Brenner Hermann (2018). Length of hospital stay and dementia: A systematic review of observational studies. International Journal of Geriatric Psychiatry.

[5] Bedford PD (1955). Adverse cerebral effects of anaesthesia on old people. Lancet Lond Engl.

[6] Symes E, Maruff P, Ajani A, Currie J (2000). Issues associated with the identification of cognitive change following coronary artery bypass grafting. Aust N Z J Psychiatry.

[7] Evered L, Silbert B, Knopman DS, Scott DA, DeKosky ST, Rasmussen LS (2018). Recommendations for the nomenclature of cognitive change associated with Anaesthesia and Surgery-20181. J Alzheimers Dis JAD.

[8] Lewis M, Maruff P, Silbert B (2004). Statistical and conceptual issues in defining postoperative cognitive dysfunction. Neurosci Biobehav Rev.

[9] Murkin JM, Newman SP, Stump DA, Blumenthal JA (1995). Statement of consensus on assessment of neurobehavioral outcomes after cardiac surgery. Ann Thorac Surg.

[10] Murkin JM, Stump DA, Blumenthal JA, McKhann G (1997). Defining dysfunction: group means versus incidence analysis--a statement of consensus. Ann Thorac Surg.

[11] Ghoneim MM, Block RI (2012). Clinical, methodological and theoretical issues in the assessment of cognition after anaesthesia and surgery: a review. Eur J Anaesthesiol.

[12] Newman S, Klinger L, Venn G, Smith P, Harrison M, Treasure T (1989). Subjective reports of cognition in relation to assessed cognitive performance following coronary artery bypass surgery. J Psychosom Res.

[13] Shaw PJ, Bates D, Cartlidge NE, French JM, Heaviside D, Julian DG (1987). Neurologic and neuropsychological morbidity following major surgery: comparison of coronary artery bypass and peripheral vascular surgery. Stroke..

[14] Newman MF, Mathew JP, Grocott HP, Mackensen GB, Monk T, Welsh-Bohmer KA (2006). Central nervous system injury associated with cardiac surgery. Lancet Lond Engl.

[15] Evered LA, Silbert BS, Scott DA, Maruff P, Laughton KM, Volitakis I (2009). Plasma amyloid beta42 and amyloid beta40 levels are associated with early cognitive dysfunction after cardiac surgery. Ann Thorac Surg.

[16] Gao L, Taha R, Gauvin D, Othmen LB, Wang Y, Blaise G (2005). Postoperative cognitive dysfunction after cardiac surgery. Chest..

[17] Newman MF, Kirchner JL, Phillips-Bute B, Gaver V, Grocott H, Jones RH (2001). Longitudinal assessment of neurocognitive function after coronary-artery bypass surgery. N Engl J Med.

[18] Pugsley W, Klinger L, Paschalis C, Treasure T, Harrison M, Newman S (1994). The impact of microemboli during cardiopulmonary bypass on neuropsychological functioning. Stroke..

[19] Selnes OA, Grega MA, Bailey MM, Pham LD, Zeger SL, Baumgartner WA (2008). Cognition 6 years after surgical or medical therapy for coronary artery disease. Ann Neurol.

[20] Moller JT, Cluitmans P, Rasmussen LS, Houx P, Rasmussen H, Canet J (1998). Long-term postoperative cognitive dysfunction in the elderly ISPOCD1 study. ISPOCD investigators. International study of postoperative cognitive dysfunction. Lancet Lond Engl.

[21] Mason SE, Noel-Storr A, Ritchie CW (2010). The impact of general and regional anesthesia on the incidence of postoperative cognitive dysfunction and postoperative delirium: a systematic review with meta-analysis. J Alzheimers Dis JAD.

[22] Silbert BS, Evered LA, Scott DA (2014). Incidence of postoperative cognitive dysfunction after general or spinal anaesthesia for extracorporeal shock wave lithotripsy. Br J Anaesth.

[23] Berger M, Schenning KJ, Brown CH, Deiner SG, Whittington RA, Eckenhoff RG (2018). Best practices for postoperative brain health: recommendations from the fifth international perioperative neurotoxicity working group. Anesth Analg.

[24] Miller D, Lewis SR, Pritchard MW, Schofield-Robinson OJ, Shelton CL, Alderson P (2018). Intravenous versus inhalational maintenance of anaesthesia for postoperative cognitive outcomes in elderly people undergoing non-cardiac surgery. Cochrane Database Syst Rev.

[25] Lu X, Jin X, Yang S, Xia Y (2018). The correlation of the depth of anesthesia and postoperative cognitive impairment: a meta-analysis based on randomized controlled trials. J Clin Anesth.

[26] Hou R, Wang H, Chen L, Qiu Y, Li S (2018). POCD in patients receiving total knee replacement under deep vs light anesthesia: a randomized controlled trial. Brain Behav.

[27] Erdogan MA, Demirbilek S, Erdil F, Aydogan MS, Ozturk E, Togal T (2012). The effects of cognitive impairment on anaesthetic requirement in the elderly. Eur J Anaesthesiol.

[28] Purdon PL, Pavone KJ, Akeju O, Smith AC, Sampson AL, Lee J (2015). The ageing brain: age-dependent changes in the electroencephalogram during propofol and sevoflurane general anaesthesia. Br J Anaesth.

[29] Ma D, Rajakumaraswamy N, Maze M (2004). alpha2-Adrenoceptor agonists: shedding light on neuroprotection?. Br Med Bull.

[30] Mo Y, Zimmermann AE (2013). Role of dexmedetomidine for the prevention and treatment of delirium in intensive care unit patients. Ann Pharmacother.

[31] Su X, Meng Z-T, Wu X-H, Cui F, Li H-L, Wang D-X (2016). Dexmedetomidine for prevention of delirium in elderly patients after non-cardiac surgery: a randomised, double-blind, placebo-controlled trial. Lancet Lond Engl.

[32] Zhou C, Zhu Y, Liu Z, Ruan L (2016). Effect of dexmedetomidine on postoperative cognitive dysfunction in elderly patients after general anaesthesia: a meta-analysis. J Int Med Res.

[33] Deiner S, Luo X, Lin H-M, Sessler DI, Saager L, Sieber FE (2017). Intraoperative infusion of Dexmedetomidine for prevention of postoperative delirium and cognitive dysfunction in elderly patients undergoing major elective noncardiac surgery: a randomized clinical trial. JAMA Surg.

[34] Chernov VI, Efimova NY, Efimova IY, Akhmedov SD, Lishmanov YB (2006). Short-term and long-term cognitive function and cerebral perfusion in off-pump and on-pump coronary artery bypass patients. Eur J Cardio-Thorac Surg Off J Eur Assoc Cardio-Thorac Surg.

[35] Paolin A, Michielon P, Betetto M, Sartori G, Valfré C, Rodriguez G (2010). Lower perfusion pressure during hypothermic cardiopulmonary bypass is associated with decreased cerebral blood flow and impaired memory performance 6 months postoperatively. Heart Surg Forum.

[36] Slater JP, Guarino T, Stack J, Vinod K, Bustami RT, Brown JM (2009). Cerebral oxygen desaturation predicts cognitive decline and longer hospital stay after cardiac surgery. Ann Thorac Surg.

[37] Abildstrom H, Høgh P, Sperling B, Moller JT, Yndgaard S, Rasmussen LS (2002). Cerebral blood flow and cognitive dysfunction after coronary surgery. Ann Thorac Surg.

[38] Goettel N, Burkhart CS, Rossi A, Cabella BCT, Berres M, Monsch AU (2017). Associations between impaired cerebral blood flow autoregulation, cerebral oxygenation, and biomarkers of brain injury and postoperative cognitive dysfunction in elderly patients after major noncardiac surgery. Anesth Analg.

[39] Gong G-L, Liu B, Wu J-X, Li J-Y, Shu B-Q, You Z-J (2018). Postoperative cognitive dysfunction induced by different surgical methods and its risk factors. Am Surg.

[40] Shoair OA, Grasso Ii MP, Lahaye LA, Daniel R, Biddle CJ, Slattum PW (2015). Incidence and risk factors for postoperative cognitive dysfunction in older adults undergoing major noncardiac surgery: a prospective study. J Anaesthesiol Clin Pharmacol.

[41] Kadoi Y, Kawauchi C, Kuroda M, Takahashi K, Saito S, Fujita N (2011). Association between cerebrovascular carbon dioxide reactivity and postoperative short-term and long-term cognitive dysfunction in patients with diabetes mellitus. J Anesth.

[42] Puskas F, Grocott HP, White WD, Mathew JP, Newman MF, Bar-Yosef S (2007). Intraoperative hyperglycemia and cognitive decline after CABG. Ann Thorac Surg.

[43] Jiang J, Dong Y, Huang W, Bao M (2017). General anesthesia exposure and risk of dementia: a meta-analysis of epidemiological studies. Oncotarget..

[44] Kim CT, Myung W, Lewis M, Lee H, Kim SE, Lee K (2018). Exposure to general anesthesia and risk of dementia: a Nationwide population-based cohort study. J Alzheimers Dis JAD.

[45] Coyle JT, Price DL, DeLong MR (1983). Alzheimer’s disease: a disorder of cortical cholinergic innervation. Science..

[46] Gómez-Isla T, Price JL, McKeel DW, Morris JC, Growdon JH, Hyman BT (1996). Profound loss of layer II entorhinal cortex neurons occurs in very mild Alzheimer’s disease. J Neurosci.

[47] Whitehouse PJ, Price DL, Struble RG, Clark AW, Coyle JT, Delon MR (1982). Alzheimer’s disease and senile dementia: loss of neurons in the basal forebrain. Science.

[48] Douchamps V, Mathis C (2017). A second wind for the cholinergic system in Alzheimer’s therapy. Behav Pharmacol.

[49] Francis PT, Palmer AM, Snape M, Wilcock GK (1999). The cholinergic hypothesis of Alzheimer’s disease: a review of progress. J Neurol Neurosurg Psychiatry.

[50] Guerrero-Muñoz MJ, Gerson J, Castillo-Carranza DL (2015). Tau oligomers: the toxic player at synapses in Alzheimer’s disease. Front Cell Neurosci.

[51] Krstic D, Knuesel I (2013). The airbag problem-a potential culprit for bench-to-bedside translational efforts: relevance for Alzheimer’s disease. Acta Neuropathol Commun.

[52] Markesbery WR (1997). Oxidative stress hypothesis in Alzheimer’s disease. Free Radic Biol Med.

[53] Selles M. Clara, Oliveira Mauricio M., Ferreira Sergio T. (2018). Brain Inflammation Connects Cognitive and Non-Cognitive Symptoms in Alzheimer’s Disease. Journal of Alzheimer's Disease.

[54] Di Nino G, Adversi M, Samolsky Dekel BG, Fodale V, Rosa G, Melotti RM (2010). Peri-operative risk management in patients with Alzheimer’s disease. J Alzheimers Dis JAD.

[55] Fernandez CR, Fields A, Richards T, Kaye AD (2003). Anesthetic considerations in patients with Alzheimer’s disease. J Clin Anesth.

[56] Perez-Protto S, Geube M, Ontaneda D, Dalton JE, Kurz A, Sessler DI (2014). Sensitivity to volatile anesthetics in patients with dementia: a case-control analysis. Can J Anaesth J Can Anesth.

[57] Baruah J, Easby J, Kessell G (2008). Effects of acetylcholinesterase inhibitor therapy for Alzheimer’s disease on neuromuscular block. Br J Anaesth.

[58] Crowe S, Collins L (2003). Suxamethonium and donepezil: a cause of prolonged paralysis. Anesthesiology..

[59] Dooley M, Lamb HM (2000). Donepezil: a review of its use in Alzheimer’s disease. Drugs Aging.

[60] Bhardwaj A, Dharmavaram S, Wadhawan S, Sethi A, Bhadoria P (2011). Donepezil: a cause of inadequate muscle relaxation and delayed neuromuscular recovery. J Anaesthesiol Clin Pharmacol.

[61] Mehaffey J. Hunter, Hawkins Robert B., Tracci Margaret C., Robinson William P., Cherry Kenneth J., Kern John A., Upchurch Gilbert R. (2018). Preoperative dementia is associated with increased cost and complications after vascular surgery. Journal of Vascular Surgery.

[62] Evered LA, Silbert BS, Scott DA, Maruff P, Ames D, Choong PF (2011). Preexisting cognitive impairment and mild cognitive impairment in subjects presenting for total hip joint replacement. Anesthesiology.

[63] Khan MA, Hossain FS, Ahmed I, Muthukumar N, Mohsen A (2013). Predictors of early mortality after hip fracture surgery. Int Orthop.

[64] Seitz DP, Gill SS, Bell CM, Austin PC, Gruneir A, Anderson GM (2014). Postoperative medical complications associated with anesthesia in older adults with dementia. J Am Geriatr Soc.

[65] Cao L, Wang K, Gu T, Du B, Song J (2014). Association between APOE epsilon 4 allele and postoperative cognitive dysfunction: a meta-analysis. Int J Neurosci.

[66] Schenning KJ, Murchison CF, Mattek NC, Kaye JA, Quinn JF (2019). Sex and genetic differences in postoperative cognitive dysfunction: a longitudinal cohort analysis. Biol Sex Differ.

[67] Liu Y, Pan N, Ma Y, Zhang S, Guo W, Li H (2013). Inhaled sevoflurane may promote progression of amnestic mild cognitive impairment: a prospective, randomized parallel-group study. Am J Med Sci.

[68] Patel D, Lunn AD, Smith AD, Lehmann DJ, Dorrington KL (2016). Cognitive decline in the elderly after surgery and anaesthesia: results from the Oxford project to investigate memory and ageing (OPTIMA) cohort. Anaesthesia.

[69] Wan Y, Xu J, Ma D, Zeng Y, Cibelli M, Maze M (2007). Postoperative impairment of cognitive function in rats: a possible role for cytokine-mediated inflammation in the hippocampus. Anesthesiology.

[70] Kawano Takashi, Yamanaka Daiki, Aoyama Bun, Tateiwa Hiroki, Shigematsu-Locatelli Marie, Nishigaki Atsushi, Iwata Hideki, Locatelli Fabricio M., Yokoyama Masataka (2018). Involvement of acute neuroinflammation in postoperative delirium-like cognitive deficits in rats. Journal of Anesthesia.

[71] Xu Z, Dong Y, Wang H, Culley DJ, Marcantonio ER, Crosby G, et al. Age-dependent postoperative cognitive impairment and Alzheimer-related neuropathology in mice. Sci Rep. 2014;4. 10.1038/srep03766. 10.1038/srep03766 PMC3895908 24441878

[72] Crosby C, Culley DJ, Baxter MG, Yukhananov R, Crosby G (2005). Spatial memory performance 2 weeks after general anesthesia in adult rats. Anesth Analg.

[73] Wu J, Zhang M, Li H, Sun X, Hao S, Ji M (2016). BDNF pathway is involved in the protective effects of SS-31 on isoflurane-induced cognitive deficits in aging mice. Behav Brain Res.

[74] Liu W, Xu J, Wang H, Xu C, Ji C, Wang Y (2012). Isoflurane-induced spatial memory impairment by a mechanism independent of amyloid-beta levels and tau protein phosphorylation changes in aged rats. Neurol Res.

[75] Callaway JK, Wood C, Jenkins TA, Royse AG, Royse CF (2016). Isoflurane in the presence or absence of surgery increases hippocampal cytokines associated with memory deficits and responses to brain injury in rats. Behav Brain Res.

[76] Xu X, Zhang Q, Tian X, Wang G (2016). Sevoflurane anesthesia induces neither contextual fear memory impairment nor alterations in local population connectivity of medial prefrontal cortex local field potentials networks in aged rats. Fundam Clin Pharmacol.

[77] Le Freche H, Brouillette J, Fernandez-Gomez F-J, Patin P, Caillierez R, Zommer N (2012). Tau phosphorylation and sevoflurane anesthesia: an association to postoperative cognitive impairment. Anesthesiology.

[78] Ji M, Dong L, Jia M, Liu W, Zhang M, Ju L (2014). Epigenetic enhancement of brain-derived neurotrophic factor signaling pathway improves cognitive impairments induced by isoflurane exposure in aged rats. Mol Neurobiol.

[79] Guo S, Liu L, Wang C, Jiang Q, Dong Y, Tian Y (2018). Repeated exposure to sevoflurane impairs the learning and memory of older male rats. Life Sci.

[80] Tang JX, Eckenhoff MF (2013). Anesthetic effects in Alzheimer transgenic mouse models. Prog Neuro-Psychopharmacol Biol Psychiatry.

[81] Bianchi SL, Tran T, Liu C, Lin S, Li Y, Keller JM (2008). Brain and behavior changes in 12-month-old Tg2576 and nontransgenic mice exposed to anesthetics. Neurobiol Aging.

[82] Tang JX, Mardini F, Caltagarone BM, Garrity ST, Li RQ, Bianchi SL (2011). Anesthesia in presymptomatic Alzheimer’s disease: a study using the triple-transgenic mouse model. Alzheimers Dement J Alzheimers Assoc.

[83] Olsson B, Lautner R, Andreasson U, Öhrfelt A, Portelius E, Bjerke M (2016). CSF and blood biomarkers for the diagnosis of Alzheimer’s disease: a systematic review and meta-analysis. Lancet Neurol.

[84] Mehta PD, Pirttilä T, Mehta SP, Sersen EA, Aisen PS, Wisniewski HM (2000). Plasma and cerebrospinal fluid levels of amyloid beta proteins 1-40 and 1-42 in Alzheimer disease. Arch Neurol.

[85] Fukumoto H, Tennis M, Locascio JJ, Hyman BT, Growdon JH, Irizarry MC (2003). Age but not diagnosis is the main predictor of plasma amyloid beta-protein levels. Arch Neurol.

[86] Fei M, Jianghua W, Rujuan M, Wei Z, Qian W (2011). The relationship of plasma Aβ levels to dementia in aging individuals with mild cognitive impairment. J Neurol Sci.

[87] Kim HJ, Park KW, Kim TE, Im JY, Shin HS, Kim S (2015). Elevation of the plasma Aβ40/Aβ42 ratio as a diagnostic marker of sporadic early-onset Alzheimer’s disease. J Alzheimers Dis JAD.

[88] Tang JX, Baranov D, Hammond M, Shaw LM, Eckenhoff MF, Eckenhoff RG (2011). Human Alzheimer and inflammation biomarkers after anesthesia and surgery. Anesthesiology.

[89] Berger M, Nadler JW, Friedman A, McDonagh DL, Bennett ER, Cooter M (2016). The effect of Propofol versus Isoflurane anesthesia on human cerebrospinal fluid markers of Alzheimer’s disease: results of a randomized trial. J Alzheimers Dis JAD.

[90] Pikwer A, Castegren M, Namdar S, Blennow K, Zetterberg H, Mattsson N (2017). Effects of surgery and propofol-remifentanil total intravenous anesthesia on cerebrospinal fluid biomarkers of inflammation, Alzheimer’s disease, and neuronal injury in humans: a cohort study. J Neuroinflammation.

[91] Xie Z, Swain CA, Ward SAP, Zheng H, Dong Y, Sunder N (2014). Preoperative cerebrospinal fluid β-amyloid/tau ratio and postoperative delirium. Ann Clin Transl Neurol.

[92] Planel E, Richter KEG, Nolan CE, Finley JE, Liu L, Wen Y (2007). Anesthesia leads to tau hyperphosphorylation through inhibition of phosphatase activity by hypothermia. J Neurosci.

[93] Xiao H, Run X, Cao X, Su Y, Sun Z, Tian C (2013). Temperature control can abolish anesthesia-induced tau hyperphosphorylation and partly reverse anesthesia-induced cognitive impairment in old mice. Psychiatry Clin Neurosci.

[94] Dong Y, Wu X, Xu Z, Zhang Y, Xie Z (2012). Anesthetic isoflurane increases phosphorylated tau levels mediated by caspase activation and Aβ generation. PLoS One.

[95] Feng C, Liu Y, Yuan Y, Cui W, Zheng F, Ma Y (2016). Isoflurane anesthesia exacerbates learning and memory impairment in zinc-deficient APP/PS1 transgenic mice. Neuropharmacology..

[96] Li C, Liu S, Xing Y, Tao F (2014). The role of hippocampal tau protein phosphorylation in isoflurane-induced cognitive dysfunction in transgenic APP695 mice. Anesth Analg.

[97] Tang JX, Mardini F, Janik LS, Garrity ST, Li RQ, Bachlani G (2013). Modulation of murine Alzheimer pathogenesis and behavior by surgery. Ann Surg.

[98] Tao G, Zhang J, Zhang L, Dong Y, Yu B, Crosby G (2014). Sevoflurane induces tau phosphorylation and glycogen synthase kinase 3β activation in young mice. Anesthesiology.

[99] Tan W-F, Cao X-Z, Wang J-K, Lv H-W, Wu B-Y, Ma H (2010). Protective effects of lithium treatment for spatial memory deficits induced by tau hyperphosphorylation in splenectomized rats. Clin Exp Pharmacol Physiol.

[100] Tang N, Jiang R, Wang X, Wen J, Liu L, Wu J (1657). Insulin resistance plays a potential role in postoperative cognitive dysfunction in patients following cardiac valve surgery. Brain Res.

[101] Kline R, Wong E, Haile M, Didehvar S, Farber S, Sacks A (2016). Peri-operative inflammatory cytokines in plasma of the elderly correlate in prospective study with postoperative changes in cognitive test scores. Int J Anesthesiol Res.

[102] Liu X, Yu Y, Zhu S (2018). Inflammatory markers in postoperative delirium (POD) and cognitive dysfunction (POCD): a meta-analysis of observational studies. PLoS One.

[103] Cui R-S, Wang K, Wang Z-L (2018). Sevoflurane anesthesia alters cognitive function by activating inflammation and cell death in rats. Exp Ther Med.

[104] Perucho J, Rubio I, Casarejos MJ, Gomez A, Rodriguez-Navarro JA, Solano RM (2010). Anesthesia with isoflurane increases amyloid pathology in mice models of Alzheimer’s disease. J Alzheimers Dis JAD.

[105] Wu X, Lu Y, Dong Y, Zhang G, Zhang Y, Xu Z (2012). The inhalation anesthetic isoflurane increases levels of proinflammatory cytokine TNF-α, IL-6 and IL-1β. Neurobiol Aging.

[106] Cibelli M, Fidalgo AR, Terrando N, Ma D, Monaco C, Feldmann M (2010). Role of interleukin-1beta in postoperative cognitive dysfunction. Ann Neurol.

[107] Zheng J-W, Meng B, Li X-Y, Lu B, Wu G-R, Chen J-P (2017). NF-κB/P65 signaling pathway: a potential therapeutic target in postoperative cognitive dysfunction after sevoflurane anesthesia. Eur Rev Med Pharmacol Sci.

[108] Acharya NK, Goldwaser EL, Forsberg MM, Godsey GA, Johnson CA, Sarkar A (1620). Sevoflurane and Isoflurane induce structural changes in brain vascular endothelial cells and increase blood-brain barrier permeability: possible link to postoperative delirium and cognitive decline. Brain Res.

[109] Zheng B, Lai R, Li J, Zuo Z (2017). Critical role of P2X7 receptors in the neuroinflammation and cognitive dysfunction after surgery. Brain Behav Immun.

[110] Ling Y, Ma W, Yu L, Zhang Y, Liang Q (2015). Decreased PSD95 expression in medial prefrontal cortex (mPFC) was associated with cognitive impairment induced by sevoflurane anesthesia. J Zhejiang Univ Sci B.

[111] Miao Huihui, Dong Yuanlin, Zhang Yiying, Zheng Hui, Shen Yuan, Crosby Gregory, Culley Deborah J., Marcantonio Edward R., Xie Zhongcong (2017). Anesthetic Isoflurane or Desflurane Plus Surgery Differently Affects Cognitive Function in Alzheimer’s Disease Transgenic Mice. Molecular Neurobiology.

[112] Zhang C, Zhang Y, Shen Y, Zhao G, Xie Z, Dong Y (2017). Anesthesia/surgery induces cognitive impairment in female Alzheimer’s disease transgenic mice. J Alzheimers Dis JAD.

[113] Li L, Li Z, Cao Y, Fan D, Chui D, Guo X (2016). Increased extrasynaptic GluN2B expression is involved in cognitive impairment after isoflurane anesthesia. Exp Ther Med.

[114] Uchimoto K, Miyazaki T, Kamiya Y, Mihara T, Koyama Y, Taguri M (2014). Isoflurane impairs learning and hippocampal long-term potentiation via the saturation of synaptic plasticity. Anesthesiology..

[115] Zurek AA, Yu J, Wang D-S, Haffey SC, Bridgwater EM, Penna A (2014). Sustained increase in α5GABAA receptor function impairs memory after anesthesia. J Clin Invest.

[116] Zhang D-X, Jiang S, Yu L-N, Zhang F-J, Zhuang Q, Yan M (2015). The effect of sevoflurane on the cognitive function of rats and its association with the inhibition of synaptic transmission. Int J Clin Exp Med.

[117] Wei H, Xie Z (2009). Anesthesia, calcium homeostasis and Alzheimer’s disease. Curr Alzheimer Res.

[118] Qi Z, Tianbao Y, Yanan L, Xi X, Jinhua H, Qiujun W (2017). Pre-treatment with nimodipine and 7.5% hypertonic saline protects aged rats against postoperative cognitive dysfunction via inhibiting hippocampal neuronal apoptosis. Behav Brain Res.

[119] Zhang Q, Li Y, Bao Y, Yin C, Xin X, Guo Y (2018). Pretreatment with nimodipine reduces incidence of POCD by decreasing calcineurin mediated hippocampal neuroapoptosis in aged rats. BMC Anesthesiol.

[120] Zhang G, Dong Y, Zhang B, Ichinose F, Wu X, Culley DJ (2008). Isoflurane-induced caspase-3 activation is dependent on cytosolic calcium and can be attenuated by memantine. J Neurosci.

